# Predictors of Extra-Marital Partnerships among Women Married to Fishermen along Lake Victoria in Kisumu County, Kenya

**DOI:** 10.1371/journal.pone.0095298

**Published:** 2014-04-18

**Authors:** Zachary Kwena, Isaac Mwanzo, Chris Shisanya, Carol Camlin, Janet Turan, Lilian Achiro, Elizabeth Bukusi

**Affiliations:** 1 Center for Microbiology Research, Kenya Medical Research Institute, Kisumu, Kenya; 2 Department of Community Health, Kenyatta University, Nairobi, Kenya; 3 Department of Geography, Kenyatta University, Nairobi, Kenya; 4 Department of Obstetrics, Gynecology and Reproductive Sciences, University of California at San Francisco, San Francisco, California, United States of America; 5 Department of Health Care Organization and Policy, University of Alabama at Birmingham, Birmingham, Alabama, United States of America; International AIDS Vaccine Initiative, United States of America

## Abstract

**Background:**

The vulnerability of women to HIV infection makes establishing predictors of women's involvement in extra-marital partnerships critical. We investigated the predictors of extra-marital partnerships among women married to fishermen.

**Methods:**

The current analyses are part of a mixed methods cross-sectional survey of 1090 gender-matched interviews with 545 couples and 12 focus group discussions (FGDs) with 59 couples. Using a proportional to size simple random sample of fishermen as our index participants, we asked them to enrol in the study with their spouses. The consenting couples were interviewed simultaneously in separate private rooms. In addition to socio-economic and demographic data, we collected information on sexual behaviour including extra-marital sexual partnerships. We analysed these data using descriptive statistics and multivariate logistic regression. For FGDs, couples willing to participate were invited, consented and separated for simultaneous FGDs by gender-matched moderators. The resultant audiofiles were transcribed verbatim and translated into English for coding and thematic content analysis using NVivo 9.

**Results:**

The prevalence of extra-marital partnerships among women was 6.2% within a reference time of six months. Factors that were independently associated with increased likelihood of extra-marital partnerships were domestic violence (aOR, 1.45; 95% CI 1.09–1.92), women reporting being denied a preferred sex position (aOR, 3.34; 95% CI 1.26–8.84) and spouse longer erect penis (aOR, 1.34; 95% CI 1.00–1.78). Conversely, women's age – more than 24years (aOR, 0.33; 95% CI 0.14–0.78) and women's increased sexual satisfaction (aOR, 0.92; 95% CI 0.87–0.96) were associated with reduced likelihood of extra-marital partnerships.

**Conclusion:**

Domestic violence, denial of a preferred sex positions, longer erect penis, younger age and increased sexual satisfaction were the main predictors of women's involvement in extra-marital partnerships. Integration of sex education, counselling and life skills training in couple HIV prevention programs might help in risk reduction.

## Introduction

Extra-marital partnerships have been associated with HIV infection in marriages and a range of psycho-social problems [1]–[5]. Marriage has consistently been reported as one of the risk factors for HIV infection partly due to extra-marital partnerships of one or both spouses in the context of low or no condom use[6], [7]. Spouses involved in unprotected extra-marital sex act as conduits through which HIV enters marriages whose partners were hitherto concordant negative. Apart from HIV and other STIs as health conditions associated with extra-marital partnerships, major adverse cardiovascular events (MACE) have also been observed for those at risk of such events[8], [9]. Cardiac deaths observed in situations of extra-marital sex are associated with psychological stress due to the need for discretion and haste during such encounters[9]. Psycho-social problems associated with extra-marital partnerships, whether actual or suspected, are many and varied. Domestic violence, depression, anger, divorce and/or even spousal homicide are among the commonest of these psycho-social problems [10], [11]. For instance, many marriage dissolutions, up to 50% in developed countries, are due to extra-marital partnership concerns[12], [13].

Despite the negative consequences of extra-marital partnerships, the behaviour is reported in many cultures across the world. Overally, it is estimated that between 30–60% of men and 20–50% of women report extra-marital partnerships in their lifetime[14]. Notwithstanding the problems of under-reporting especially among women due to cultural constraints[15], the prevalence of extra-marital partnerships in sub-Saharan Africa appear to be much higher in men compared to women[16], [17]. As such, existing studies on extra-marital partnerships in sub-Saharan Africa have mainly focused on men while treating women in marriages merely as victims[18]–[21]. Even though the prevalence of extra-marital partnerships may be lower among women, concomitant effects may be equal or greater than men given women's biological and socio-cultural vulnerabilities[22]. Thus, there is urgency in identifying and addressing factors that predispose women to extra-marital partnerships.

Many factors have been identified as predictors of men's involvement in extra-marital partnerships. Most of these factors relate to intrapersonal, interpersonal and contextual attributes[23], [24]. The common intrapersonal factors that have been isolated as predictors of extra-marital sex include: age, gender, religiosity, education and income levels while the interpersonal factors commonly cited are sexual and marital satisfaction, marital commitment and partner attachment levels and; the main contextual factor is travelling and physical separation[2], [25]. Since studies that cited these factors as predictors of extra-marital partnerships in sub-Saharan Africa are focused mostly on men, there is need for similar studies with focus on married women in sub-Saharan Africa.

Married couples in Kenya as elsewhere in sub-Saharan Africa have been observed to engage in extra-marital partnerships which may increase their risk of HIV infection especially in the context of low or no condom use within marriage [26], [27]. The practice of polygamy that is common in this region may further complicate the issue extra-marital partnerships especially among more egalitarian women. Extra-marital partnerships have been more pronounced in groups classified as ‘key populations’ for HIV infection that include truck drivers, migrant workers, sex workers and fisherfolk in communities such as those living along Lake Victoria. More importantly, fishing communities along Lake Victoria are reported to commonly engage in high risk sexual behaviour and have high HIV prevalence rates of up to 26.5% compared to the regional and Kenyan national average of 15.1% and 5.6%, respectively [28]–[30].

Understanding predictors of extra-marital sex among women is critical in designing prevention interventions that address risky sexual behaviour, the likely marital HIV infections as well as other associated negative consequences such as domestic violence, divorce and spousal homicides. Although numerous studies have been designed to identify predictors of men's involvement in extra-marital partnerships, few studies have focused on women. Thus, this paper seeks to fill this gap by identifying predictors of extra-marital partnerships that place women and their spouses at increased risk for HIV infection.

## Methods

### Ethical approval

The study was approved by the Kenya Medical Research Institute (KEMRI) Ethical Review Committee under SSC No. 1989. All participants gave written informed consent before participation in the study.

### Design and settings

This was part of a larger cross-sectional mixed methods study conducted among 604 married couples from fishing communities on Lake Victoria of Kisumu County, Kenya between September 2011 and June 2012 to establish the prevalence and determinants of couple sexual concurrency. This paper aims to determine the predictors of extra-marital partnerships among married women using structured interview data and to put these findings into context using focus group discussion data. Detailed description of the methods used in this study have been given elsewhere[31].

### Structured interview data

#### Sampling and data collection

We conducted a total of 1090 structured interviews with 545 married couples from 33 beaches. On each beach, we first obtained a list of all fishermen and then with the help of beach management officials made a second list of fishermen who they knew or suspected were married and between ages 18–45; this minimized our screen-to-enrol ratio (see Kwena and colleagues for details[31]). Individual fishermen were randomly selected from the list of married fishermen on each beach. Marriage in this context was defined as any two people of the opposite sex who have lived together in a union sanctioned by state-mandated authorities as husband and wife for at least three months. The selected fishermen were approached and asked if they were willing to participate in a study that enrolled couples. Willing couples were asked to come to the study clinic for study-related procedures that included eligibility screening, consenting and face-to-face interviews. The interview covered a number of topics that included: socio-economic and demographic details, marital and sexual relationships and number of sexual partners in six months preceding the study. For those who had sexual partners beyond the spouse in the preceding six months, we asked details of the extra-marital partners including the type, end dates of the relationship and if the relationship was on-going.

#### Measurements

Our outcome variable was extra-marital sex which we operationalized as any sexual relationship(s) developing after and running parallel to marital relationships. We categorized women into two groups: women reporting extra-marital sex in the preceding six months and those not involved in extra-marital sex over the same reference period. We considered the following key predictor variables based on our theoretical convictions and well as existing literature:

#### Socio-demographic and economic

We directly asked participants for information on various socio-demographic and economic variables. The variables on which we collected information on included: current age, level of education, occupation, type of marriage (whether monogamous or polygamous), physical separation from spouse, previous HIV test result disclosure.

#### Domestic violence, male dominance sex roles and sexual satisfaction

We described in details elsewhere how we constructed measures of domestic violence, male dominant sex roles and sexual satisfaction[31]. In brief, domestic violence scale was created from 9 items that had yes/no responses taken from demographic and health survey questionnaires[32] with Cronbach alpha of 0.61 for men and 0.78 for women. The nine items measured actual experience of violence within the households based on the way questions were framed. For this scale, we asked *‘does your spouse do or do you ever do any of the following things to your partner? Slap you? (Slap her?); Kick, drag or beat you up? (Kick, drag or beat her up?)’*. The scale ranged from 0–9 where zero score indicates lower perpetuation of domestic violence and 9 higher perpetuation of domestic violence. Male dominance in sex roles and sexual satisfaction scales were adapted and modified from commonly used psychometric measures [33], [34]. Male dominance sex roles scale included eight items such as ‘*men cannot live with just one woman*’ and ‘*only a bad girl shows that she likes sex a lot*’ scored on a four-point Likert-type scale with Cronbach's alpha of 0.54 for men and 0.53 for women. The scale ranged from 0–24 where zero indicates endorsement of respect for women in a relationship and 24 indicates male dominance and disrespect of women in sex roles. As such, higher scores on this scale reflect endorsement of sexual standards that disrespect women. Sexual satisfaction scale, on the other hand, included 25 items such as ‘*I feel that my partner enjoys our sex life*’ and ‘*our sex life is monotonous*’ scored on a four-point Likert-type scale with Cronbach alpha of 0.72 for men and 0.86 for women. The scale ranged from 0–75 where zero indicates lower sexual satisfaction and 75 show greater sexual satisfaction. Higher scores on this scale indicate stronger sexual satisfaction with the spouse.

#### Penile size –

We included this variable because of the interest and controversies that surround men's pursuant of penile enlargement ostensibly to sexually satisfy their spouses while women do not necessarily approve of it[35]. Penis size was measured through self-report by both men and women. Using a 15-inch ruler as a guide, we asked men to estimate their fully erect penis size and we asked women to estimate their spouses fully erect penis. Since these were perceived estimates from the two spouses, the observed discrepancies were resolved by taking an average of the two responses obtained. For categorical variables, the study examined the responses from the two partners logically and opted to go with the response from a partner who might have had no apparent reason to mislead. For instance, the study asked both partners the number of wives the man had. If the woman reported two and the man one, the study considered the woman's response as correct because of desirability bias that may be inherent in the men's response on this issue.

#### Data analysis

Data was entered on-site in CSPro 4.0 that allows in-built logical checks and skip patterns before being imported into SPSS 18 (Version 18.0, SPSS Inc., Chicago, IL) for cleaning and analysis. We used both descriptive and inferential – mainly logistic regression – methods to arrive at conclusions. After the descriptive analyses, we conducted a series of bivariate analyses testing the association of one explanatory variable at a time with the outcome which is women's extra-marital relationships in the past six months. This was essential in order to shortlist variables for multivariable analysis since we had a large number of independent variables. We also used this step to only shortlist variables at p≤0.25 level of significance for multivariate analysis. This inclusion level was chosen as the most conservative value in practice that gives a chance to each variable that may not be significant at the usual 0.05 significance level but may become significant in the modeling process jointly with other variables [36]. The explanatory variables were then entered into the multivariate logistic model directly and resultant adjusted odds ratios and their 95% confidence interval reported. We used the ‘enter’ method of the multivariate logistic model which simultaneously enters all variables into the model as a block and assess the contribution of each to the outcome variable.

### Focus group discussions

#### Sample size and sample selection

For the 12 focus group discussions conducted, six were with either gender. Ten of the 12 FGDs had 10 participants each and 2 had 9 participants. The participants in the FGDs and face-to-face interviews were mutually exclusive such that no couple participated in both. The flexibility of focus groups as qualitative method enabled us to evoke meaningful and culturally salient responses and thus helped uncover entrenched issues pertaining to extra-marital partnerships in this fishing community to supplement quantitative data. The number of FGDs was determined by saturation point, which is a point where no new information is forthcoming on the various themes we were pursuing. All the 33 fishing beaches within Kisumu County participated and we made purposeful efforts to include married couples from all the beaches. Participants were identified in collaboration with the beach management units during the community mobilization and preparation for the study. The FGD guide was developed in English and translated and back-translated in to the local language (Dholuo) for verification. Fishermen, who were over 18 years old, married and knowledgeable of the fishing community, were our index participants. Similar to structured interview participants, FGD participants were approached, briefed about the study and asked whether they were willing to be recruited in the study with their spouses. Those willing were asked to inform their spouses about the study and find out if they were also interested. The interested couples were then invited to come for focus group discussions.

#### Consenting and data collection

When the couples invited arrived at the study clinic, they were given the full details about the study and consented together as a couple before being separated into different rooms for group discussions. The group discussions for the separated spouses took place concurrently to avoid partners influencing each other's responses during the discussions. For confidentiality purposes, partners of a couple were given same number tag that the moderator called out each time they wanted to speak instead of their names. The number tags also helped us to link views from the same couple on different issues. The discussions were recorded by voice-activated digital recorder and later uploaded into password-protected folders on the study computer. On average, the focus group discussions took 1.5 hours. The discussions were conducted by gender-matched moderators with the themes deliberately limited to cover group normative behaviour and diversity of views in the group. We covered a wide range of issues which included courtship and marriage in fishing communities, relationship and sexual satisfaction, extra-marital relationships, consequences of extra-marital relationships and possible interventions to reduce extra-marital relationships in the fishing communities. All FGDs were conducted in Dholuo and the resultant audiofiles transcribed verbatim and later translated into English by a trained transcriptionist.

#### Data analysis

Preliminary data analysis and preparation started and continued in tandem with data flow from the field. We scanned through the transcripts as they became available to develop broad codes and eventually fine codes. The broad codes represented the thematic areas that our study was focused on under which we had several fine codes. Analysis of the transcripts followed grounded theory tenets that allow analytical themes to emerge from voices of participants. Specifically, we analyzed data by constant comparative method which is an inductive analysis derived from grounded theory[37]. Under this method, one piece of data, for instance, one interview or one statement or one theme, is taken and compared to all other pieces of data that are either similar or different to identify what makes the piece of data different or similar to other pieces of data. This helps in defining sub-themes during the process of transcripts reading, exploration and coding responses. Using NVivo 9 (QSR International Pty Ltd, Melbourne, Australia) qualitative data analysis software, we coded the transcripts, categorizing the data into broad codes (themes) and in each identified fine codes (subthemes).Coding reports were discussed in a series of meetings held among the lead author and the study staff to refine the coding framework. For this paper, we captured the following broad codes relating to sexual concurrency from the transcripts: (a) marital relationships, (b) attributes of sexual satisfaction and, (c) the role of penis size in women's extra-marital partnerships.

## Results

A total of 545 women married to fishermen were enrolled to participate in the structured interviews with a median age of 24 (IQR 21–28) mostly (86.0%) with primary level education. The majority of the women (56.7%) were affiliated to African Independent Churches and a quarter attended Protestant Churches (Table 1). Slightly more than a quarter of the women (26.4%) worked either as fish traders or brokers with over 40% describing themselves as homemakers. Over 90% of the women were in monogamous marriage; the majority of those who were in polygamous marriages were in unions that consisted of one man and two wives. A quarter of the women (23.8%) reported that their spouses were circumcised and 46.9% of the women were currently on contraceptives. The women had a median age of 24 (interquartile range [IQR], 21–28) with 25% reporting sexual debut before age 13 and a median age at first marriage of 18 (IQR, 16–20). The median duration of marriage was 6.2 years (IQR, 2.7–11.6) and the median number of children with their current spouses was 2 (IQR, 1–3). The median monthly income for the women was USD 18.4 with a quarter reporting no income at all. However, the women reported a median monthly household expenditure of USD 77 (IQR, 60–98) (Table 1).

**Table 1:** Socio-economic and demographic characteristics of women married fishermen along Lake Victoria in Kisumu County.

Characteristic (categorical)	Frequency	Percent
Level of education		
Primary	461	86.0
Post-primary	75	14.0
Religion		
Protestant	135	25.0
Catholic	99	18.3
African independent churches	306	56.7
Occupation		
Fish trader	144	26.4
Homemaker	221	40.6
Other	180	33.0
Polygamy status		
Monogamous marriage	494	90.6
Polygamous marriage	51	9.4
Spouse circumcision status		
Not circumcised	295	76.2
Circumcised	92	23.8
Use of modern family planning methods		
Not currently using	206	53.1
Currently using	182	46.9

Characteristic (continuous)	Median	IQR
Age	24	21–28
Age at sex debut	15	13–16
Age at first marriage	18	16–20
Length of current marriage (months)	75	33–139
Total number of biological children	2	1–3
Children with current spouse	2	1–3
Monthly income (USD)	18.4	0–46
Monthly household expenditure (USD)	77	60–98

Overall, 6.2% of the women reported extra-marital sex in the preceding six months. Thirty one women (5.7%) had previously been married and 39.4% reported staying apart with their current spouses for a median of three months (IQR, 2–4) in a year. Twenty seven percent of the women suspected their spouses to be involved in extra-marital relationships. These women who did suspect their spouses to be involved in extra-marital partnerships were more likely to be involved in extra-marital partnerships themselves than those who did not (p<0.01) (Table 2). While 22.4% of the women reported being denied sex at least once in the preceding month, 9.4% reported that they were denied preferred sex position by their spouses. Women who reported extra-marital partnerships were more likely to have been denied either sex or preferred sex position. One quarter (73.8%) of the women achieved orgasm during the most recent sex with their spouses and 65.7% reported to have ever used condoms. A slightly higher proportion of those involved in extra-marital partnerships reported ever using condoms (73.5%) compared to those who were not involved in extra-marital partnerships (65.2%). Women reported a median number of sex days per week of 2 (IQR, 2–3) and a half said that they took 15 minutes to reach climax (IQR, 5–30). Women's spouses reported a median fully erect penis size of 5 inches (IQR, 5–6) with spouses of women involved in extra-marital sex reporting longer fully erect penis size (6 inches) compared to those not reporting extra-marital sex. Women's median sexual satisfaction score was 78 with those reporting extra-marital sex having lower scores compared to those who were not (p<0.01). The median scores on men's domestic violence and male dominant sex script were 2 (IQR, 1–3) and 10 (IQR, 7–13), respectively. Women whose spouses scored highly on the two scales were more likely to report extra-marital partnerships.

**Table 2:** Relationship and sexual attributes by extra-marital partnership status among women married to fishermen along Lake Victoria in Kisumu County.

Categorical variables	Overall (n = 545)		Not involved in extra-marital sex (n = 511)		Involved in extra-marital sex (n = 34)		p-value
	Freq	percent	Freq	percent	Freq	percent	
Involved in extra-marital sex	34	6.2	n/a	n/a	n/a	n/a	n/a
Previously married	31	5.7	28	5.5	3	8.8	0.43
Physically separated from spouse part of the year	215	39.4	200	39.1	15	44.1	0.59
Suspects spouse of involvement in extra-marital sex	148	27.2	130	25.4	18	52.9	0.01
Denied sex in the preceding month	119	22.4	99	21.3	9	30.2	0.03
Denied preferred sex position	51	9.4	41	8.0	10	29.4	0.01
Achieved orgasm at last sex	402	73.8	384	75.1	18	52.9	0.01
Ever used condoms	358	65.7	333	65.2	25	73.5	0.36
**Continuous variables**	**Median**	**IQR**	**Median**	**IQR**	**Median**	**IQR**	**p-value**
Number of days had sex per week	2	2–3	2	2–3	2	2–3	0.91
Spouse time to orgasm	15	5–30	15	5–30	12	5–30	0.97
Spouse fully erect penis size (inches)	5	5–6	5	5–6	6	5–6	0.16
Women's sexual satisfaction score	78	72–84	78	72–85	72	67–78	0.01
Men's domestic violence score	2	1–3	2	1–3	3	2–4	0.01
Men's male dominance script score	10	7–13	10	7–13	12	8–15	0.03

Freq – frequency; n/a – not applicable; IOR – interquartile range.

At bivariate level, we screened for all factors with theoretical plausibility or have been shown in literature to be associated with extra-marital partnerships. Table 3 shows all the factors that we screened, those that met our cut-off criteria of p≤0.25 and those that we included in our final multivariate logistic regression model. At multivariate level, the factors that we found independently associated with increased likelihood of extra-marital partnerships were: men's increasing score on perpetuation of domestic violence (aOR, 1.45; 95% CI 1.09–1.92), women reporting being denied preferred sex position by their spouses (aOR, 334; 95% CI 1.26–8.84) and spouse longer fully erect penis size (aOR, 1.34; 95% CI 1.00–1.78). Conversely, women's older age – more than 24years (aOR, 0.33; 95% CI 0.14–0.78) and women's score on sexual satisfaction with the spouse (aOR, 0.92; 95% CI 0.87–0.96) were associated with reduced likelihood of extra-marital partnership (Table 3).

**Table 3:** Predictors of women's involvement in extra-marital partnerships among married couples in the fishing communities along Lake Victoria in Kisumu County.

Characteristics	Bivariate				Multivariate			
	p-value	OR	95% CI		p-value	aOR	95% CI	
			Lower	Upper			Lower	Upper
Age								
Youthful (≤24 years old)	Ref							
Elderly (> 24 years old)	**0.01**	**0.35**	**0.16**	**0.76**	**0.01**	**0.33**	**0.14**	**0.78**
Religion#								
Independent churches	Ref							
Main stream Christian churches	0.80	0.90	0.39	2.05	N/A			
Education level								
Primary	Ref							
Post-primary	**0.09**	**0.17**	**0.02**	**1.12**	0.18	0.25	0.03	1.95
Number of children with current spouse#	0.33	0.89	0.71	1.12	N/A			
Occupation								
Does not work in fishing industry	Ref							
Works in the fishing industry	**0.05**	**0.35**	**0.12**	**1.02**	0.18	0.46	0.14	1.45
Monthly income#								
Has no monthly income	Ref							
Has monthly income	0.61	1.24	0.55	2.80	N/A			
Polygamy status								
Monogamous marriage	Ref							
Polygamous marriage	**0.21**	**0.28**	**0.04**	**2.09**	0.21	0.25	0.03	2.21
Dowry payment#								
Dowry not paid	Ref							
Dowry paid	0.79	0.91	0.44	1.86	N/A			
Duration of marriage#	0.47	1.02	0.96	1.09	N/A			
Previous marriages#								
Married before	Ref							
Not married before	0.42	0.60	0.17	2.08	N/A			
Physical separation								
No physical separation	Ref							
Physical separation	**0.18**	**1.01**	**0.99**	**1.04**	0.91	1.05	0.48	2.30
Duration of physical separation per year (months) #	0.47	1.29	0.64	2.62	N/A			
Mobile women in preceding month#								
Not mobile in preceding month	Ref							
Mobile in preceding month	0.72	1.14	0.56	2.33	N/A			
Results of previous HIV test#								
Negative	Ref							
Positive	0.36	0.50	0.12	2.17	N/A			
HIV disclosure#								
Does not know partners HIV status	Ref							
Knows partner's HIV status	0.40	0.74	0.36	1.51	N/A			
Denied sex in preceding month								
Never denied sex in preceding month	Ref							
Denied sex in preceding month	**0.02**	**2.28**	**1.11**	**4.71**	0.61	1.26	0.52	3.06
Sex position								
Neither denied preferred sex position	Ref							
Denied preferred sex position	**<0.01**	**4.78**	**2.14**	**10.67**	**0.01**	**3.34**	**1.26**	**8.84**
Frequency of sex in a week#	0.74	0.92	0.58	1.48	N/A			
Penis size (inches)	**0.05**	**1.27**	**1.00**	**1.62**	**0.05**	**1.34**	**1.00**	**1.78**
Women's sexual satisfaction score	**<0.01**	**0.92**	**0.88**	**0.96**	**0.01**	**0.92**	**0.87**	**0.96**
Spouse's sexual satisfaction score	**0.01**	**0.94**	**0.90**	**0.98**	0.08	0.95	0.90	1.01
Spouse's perpetuation of domestic violence score	**0.01**	**1.37**	**1.08**	**1.73**	**0.01**	**1.45**	**1.09**	**1.92**
Spouse's male dominant sex role score	**0.04**	**1.09**	**1.01**	**1.18**	0.33	1.05	0.96	1.15

# Not included in the multivariate logistic regression model due to failure to meet set cut-off point of p≤0.25; N/A  =  Not applicable.

Our results show that every one unit score on men's perpetuation of domestic violence scale is associated with 45% increased likelihood of women having extra-marital partnerships. Similarly, women who were denied their preferred sex positions by their spouses were four times more likely to report extra-marital partnerships compared to their counterparts. This was corroborated and emphasized by qualitative data where during one of the focus group discussions one woman said:

*“I think that it depends on what you can do on the bed. So many sexual styles [positions] have come up. You give him a style that satisfies and if it overwhelms him, it will now be up to him. This will enable you to get satisfaction as well”* FGD#5 with women

The above quote shows that women care a lot about sex positions and styles that make them achieve sexual satisfaction. Thus, sex position was brought out by most women as very critical in women's sexual gratification. As important as this may be for women, most of them fear suggesting or discussing sex position or sexual matters with their partners. This is because they fear their suggestion being construed to mean that they have been unfaithful; particularly since couples are not used to discussing the issue of how sex is conducted with their spouses. Surprisingly, spouse longer fully erect penis was associated with increased likelihood of the women having extra-marital partnerships. From these results, every one inch longer penis increased the likelihood of women being involved in extra-marital partnership by almost one-and-half times. Similarly, our qualitative data also support this finding. Women associated large penises with pain and discomfort during sex which precludes the enjoyment and sexual satisfaction that women are supposed to feel. On this issue one woman said:

*“…some penis may be large yet my vagina is small, when he tries to insert it inside, it hurts so much that I will have to look for another man who has a smaller one [penis] and can do it in a way I can enjoy”* FGD#5 with women.

According to the women, the right penis is the size of:

*“The 15 cm ruler can make a better size. The ruler that fits a set, do you think it is a bad size? [All] It*'*s good”*. FGD#1 with women

Older women above age 25 were associated with 28% reduced likelihood of engaging extra-marital partnerships compare to younger women. This was also evident in qualitative data as shown in the quote below:

*“We should get wives who are our age mates. This is true. For example, a man who is 80 years old should not marry a 14 year old girl because there will be misunderstandings. The girl may not provide enough food for the man and the man may also not satisfy the girl on bed leading to unfaithfulness”* FGD#4 with men.

The argument that was advanced is that when the age different between the spouse is so big, satisfying each other's sexual needs becomes problematic leading to unsatisfied partner engaging extra-marital partnerships. This is further empirically confirmed by the clear relationship between lower sexual satisfaction score and extra-marital partnerships. Our data demonstrated that higher score on women's sexual satisfaction scale was associated with reduced likelihood of women reporting extra-marital partnerships. For instance, for each one unit score increase on the sexual satisfaction scale was associated with 92% reduced likelihood of women engaging in extra-marital sex. This was also supported and contextualized by qualitative data as summarized in the quote below:

*“Some [men] just take a minute and leaves you there when you are still ‘hanging’… You cannot even tell if this thing is over or still continuing. Sometime we aren*'*t satisfied yet we can*'*t explain it [to our partners]. However, when we get men who can satisfy us, we do not waste such chances. For a woman to be ready and get sexual satisfaction usually takes time. Yet he has some high sexual desire and can just finish very fast before you understand how. We are left wondering and can be very happy if we can get someone who can do it better and makes you feel that your body is satisfied. I can just continue with him because his sex is sweet and your husband can then do it on short time basis”* FGD#1 with women.

This quote contextualizes women's perspective of sexual satisfaction which may be pegged on the duration of the sexual activity; highlighting the gender differences in sexual preparation and readiness. A miss-match in the timings compromises sexual satisfaction that may lead to temptations of extra-marital partnerships.

## Discussion

We set to determine the predictors of extra-marital partnerships among women married to fishermen. We found extra-marital partnerships prevalence of 6.2% among these women. Domestic violence, sex positions, penis size, age and sexual satisfaction were associated with women's involvement in extra-marital partnerships. More specifically, we found perpetuation of domestic violence, being denied preferred sex position by the spouse, longer fully erect penis, being younger than 25 years and lack of sexual satisfaction increased the likelihood of women being involved in extra-marital partnerships. We did not find any association between women's physical separation from their spouses and their involvement in extra-marital partnerships.

Domestic violence has widely been associated with women's increased vulnerability to HIV infections[38]. Studies consistently show that women who experience domestic violence are more likely to become HIV infected compared to their counterparts. This is probably because men who abuse their wives also exhibit riskier sexual behaviours [3], [39], [40]. In the context of this study, it seems like women who experience violence from their spouses engage in extra-marital partnerships. It can also be the reverse that women who report extra-marital relationships experience domestic violence especially if their spouses suspect or get to learn about the relationships. This is even more plausible in the light of findings by Hatcher and colleagues that one of the relationship triggers of domestic violence is real or perceived extra-marital partnerships[41]. However, the cross-sectional nature of this study may not be able to determine whether extra-marital relationships or domestic violence comes first. Achieving desirable sex position during sexual intercourse is important in reaching sexual gratification for women[9], [42], [43]. Women who are not stimulated by being offered certain preferred sex positions may go an extra step to find preferred positions in which their glans are sufficiently stimulated. As reported by Haavio-Mannila and Kontula, the use of versatile sex positions is associated with women's sexual satisfaction lack of which may lead to extra-marital partnerships[43]. In this study, we find that being denied a preferred sex position is associated with women's involvement in extra-marital relationships.

Our results show that in the perspective of women, large-sized penises are associated with extra-marital partnerships possibly due to pain and discomfort during sex. This is contrary to men's perspectives that have caused them to do all sorts of things to augment their penis sizes [44]–[46]. Some have even insisted on penile enhancement against medical advisories that their penises are within the normal size [47]. This is probably because of the received and popularized fallacy equating a larger penis size with masculinity [35]. This thinking needs to be progressively corrected, especially in the light of results such as this from women who are supposedly the main beneficiaries of penile enhancement outcomes. If results from this study are anything to go by, it may be counterproductive for men to seek penile augmentation while their sexual partners are running away from the same and seeking sexual satisfaction elsewhere.

Sexual satisfaction that encompasses various aspects of sexual tact and performance is an important predictor of women's involvement in extra-marital partnerships. This is a finding that is in resonance with a number of other studies that have looked at this issue [48]–[50]. Traditionally, many sub-Saharan African societies had mechanisms for mentoring young people on their marital and family life obligations that averted many family and marital conflicts [51], [52]. While young women had their grandmothers and aunts to learn from, young men had grandfathers to learn from at dusk after work. The young people were taught how to conduct themselves and take care of partners' needs including sexual needs for stable successful marriages. However, due to urbanization and hassles of modern life, the current structures do not support this mentorship[51], [53]. Thus, many marital problems such as extra-marital partnerships being experienced may be associated with the collapse of these traditional institutions without alternatives. In Uganda, there have been attempts to re-introduce the senga (father's sister) system to try and fill the gap[54]. The senga system was a traditional channel of communication about sexual behaviour of female youth. As elaborated by Larson and colleagues, open communication and feedback mechanisms between spouses is one of the factors that enhance sexual satisfaction[55]. However, women are still bound by culturally handed down fear of discussing or giving feedback on the matters of sex [51]. This scenario makes it difficult for spouses to benefit from sexual feedback given to improve on their performance for maximum sexual satisfactions.

This study had several limitations. Our definition of married couples was based on more qualitative attributes such as both families having sanctioned the marriage and we did not require legal proof from the couples. Due to interest generated by our pre-study mobilization and community engagement activities, it is possible that some couples who were ineligible because of being single may have inaccurately reported their marital status in order to be enrolled. We tried to the extent possible to screen out those posing as married couples. Even though our participant selection was as random as possible, there still existed potential for selection biases among those who agreed to participate compared to the few who declined. It is possible that those who agreed to participate were in more stable successful marriages. Another limitation associated with studies such as this one that collect sensitive information such as women's extra-marital partnerships and ask retrospective questions are social desirability and recall biases. Women in these contexts might have tended to under-represent behaviours, especially the issue of extra-marital partnerships, to conform to community expectations [5]. Even though we reassured couples of complete privacy and confidentiality, it is possible some would still have felt uncomfortable to disclosing sensitive information for fear of their spouses coming to know how they responded. This factor may have exacerbated the potential for social desirability bias in the reporting of the number of extra-marital partners among women in this study. As with all other cross-sectional studies, we cannot establish causality. For instance, it is not possible to know whether domestic violence that is associated with extra-marital partnerships in this study preceded extra-marital partnerships or whether the reverse is true. The chronological sequence of this relationship can only be established in a purposefully designed longitudinal study.

Despite these limitations, the study is a great contribution to literature in understanding the predictors of these women's extra-marital partnerships from their own perspective. Additionally, the triangulation of quantitative and qualitative data collection methods that complement each other to put issues in context makes this study ideal to investigate the sensitive topic of sexual relationships and satisfaction. In conclusion, we found extra-marital partnerships prevalence of 6.2% with domestic violence, denied sex positions, large penis size, younger age and lack of sexual satisfaction being the main predictors of women's involvement in extra-marital partnerships. We recommend integration of sex education, marital counseling and life skills training in HIV prevention programmes as part of a package geared towards shared decision-making and open communication on matters of sex and risk reduction.
